# Differential contribution of frugivorous birds to dispersal patterns of the endangered Chinese yew (*Taxus chinensis*)

**DOI:** 10.1038/srep10045

**Published:** 2015-05-05

**Authors:** Ning Li, Shu-bo Fang, Xin-hai Li, Shu-qing An, Chang-hu Lu

**Affiliations:** 1 Laboratory of Plant–Animal Interactions, College of Biology and the Environment, Nanjing Forestry University, Nanjing 210037, China; 2 School of Life Science, Nanjing University, Nanjing 210046, China; 3 Fisheries and Life Science School, Shanghai Ocean University, Shanghai 201306, China; 4 Institute of Zoology, Chinese Academy of Sciences, Beijing 100101, China

## Abstract

The contribution of forest generalists and specialists to the dispersal pattern of tree species is not well understood. Specialists are considered low-quality dispersers because their dispersal distance is often short. However, disregard for seed deposition site may result in underestimation of the dispersal quality of specialists. The present study estimated the contribution of generalist and specialist species to the dispersal patterns of the endangered Chinese yew (*Taxus chinensis*) in a subtropical patchy forest in Southeast China. A relatively diverse assemblage of frugivorous birds visited *T. chinensis* source trees, and specialist *Hypsipetes leucocephalus* and generalist *Urocissa erythrorhyncha* were by far the highest-quantity dispersers. Considering dispersal effectiveness, the quantity aspect of effectiveness differed between the specialist assemblage and generalist assemblage; the contribution of specialists to the quantity part of effectiveness was significantly higher than that of generalists despite the relatively low diversity of specialists. After foraging, both specialist *H. leucocephalus* and generalist *U. erythrorhyncha* significantly contributed to the number of seedlings, and their contributions to seedling recruitment did not differ with regard to quality. Our results highlight the ability of *T. chinensis* to recruit an effective disperser assemblage in patchy habitats, thus increasing its persistence in this disturbed habitat.

Many plant species depend on animals for seed dispersal. Most (approximately 90%) woody plant species in tropical forests produce fruits that are dispersed by animals, particularly birds and mammals^1^. For many bird-dispersed plant species, the patterns of seed movement away from the mother plant generate templates for plant recruitment in patchy habitats^2^,^3^. Bird dispersers often differ in their contributions in to the quantity and quality aspects of dispersal effectiveness^4^,^5^. The quantity aspect is determined by the number of bird disperser visits to the seed source and the number of seeds dispersed per visit, whereas the quality aspect is determined mainly by seed treatment during transport and location of seed deposition^5^,^6^. In this respect, most theoretical and empirical studies on the process of dispersal have traditionally outlined the role of a single species or multiple disperser species as a prominent feature of this process^7^,^8^,^9^,^10^. However, an increasing amount of scientific evidence strongly supports the idea that generalization and specialization are more frequent in disperser assemblages than previously thought, with most plant species interacting with both forest generalists and forest specialists in patchy habitats^11^,^12^,^13^. Given the inherent difficulty in comparing the contributions between generalists and specialists, empirical evidence supporting the generality of the dispersal effectiveness is limited^7^,^13^. Therefore, further studies are required to elucidate the contributions to dispersal effectiveness for forest generalists and specialists in patchy habitats.

The Chinese yew (*Taxus chinensis*) is a typical relic plant species that is endemic to China. It has been listed as endangered (EN) by the IUCN^14^ and as a first-class national protected plant in China because of low pollination rates, seed predation, weak competitive ability of seedlings, and scarcity of microhabitats for recruitment^15^,^16^,^17^. Previous studies have documented that the seeds of *T. chinensis* are dispersed by birds, ants and rodents, and that birds are the most important dispersal agents for plants^16^,^17^. Here, we assess the dispersal contributions of bird species that are forest specialists or generalists for *T. chinensis*. We examined the following factors: (1) differences between forest specialists and generalists in fruit consumption among individual *T. chinensis*, and resulting effects on the quantity of seeds dispersed, and (2) the ways in which post-foraging perching among forest specialists and generalists affects their quality of seed dispersal.

## Results

### Diversity of forest generalists and specialists

During the fruiting season, we observed 28 forest generalist species (*n* = 304 individuals, 4 orders with 16 families) and 18 specialist species (*n* = 300 individuals, 3 orders with 10 families) in the patchy habitat. Species composition and diversity differed between forest generalists and specialists (Table 1). There were fewer dominant generalist species than specialist species, whereas frugivore species richness and abundance were lower in specialists than in generalists (Table 1; Table S1).

### Quantity of seed dispersal by generalists and specialists

We performed 400 h of tree observations and recorded 299 visits of 13 frugivorous bird species (8 specialist species and 5 generalist species) in 2011 and 368 visits from 19 species (11 specialist species and 7 generalist species) in 2012. The visiting frequency did not differ significantly between years (*t*-tests, *p *> 0.05), but frugivore species composition differed. More types of specialist species foraged the seeds than generalist species. In 2011, the dominant frugivore species were Black Bulbul (*Hypsipetes leucocephalus*, 151 visits), Red-billed Blue Magpie (*Urocissa erythrorhyncha*, 22 visits), and Grey-chinned Minivet (*Pericrocotus solaris*, 43 visits). In 2012, *H. leucocephalus* maintained its dominant role of consuming seeds (253 visits), but the other main foragers observed were different from those previously reported. The chestnut Bulbul (*Hemixos castanonotus*, 22 visits) and Red-whiskered Bulbul (*Pycnonotus jocosus*, 32 visits) were the main foragers in 2012 (Table 2).

During the fruiting season, 10 bird species (five generalists and five specialists) dispersed seeds following 277 visits in 2011, and 10 species (four generalists and six specialists) dispersed seeds from 346 visits in 2012. The composition of disperser species differed among generalists and specialists and the visiting frequency of specialists was significantly higher than that of generalists in both years (*t*-tests, *p* = 0.01). Moreover, *Hypsipetes leucocephalus* was the most important specialist species with regard to the quantity aspect of dispersal effectiveness in both years, whereas *U. erythrorhyncha* was the most important generalist disperser species (Table 2).

### Quality of seed dispersal by generalists and specialists

**Figure 1:**
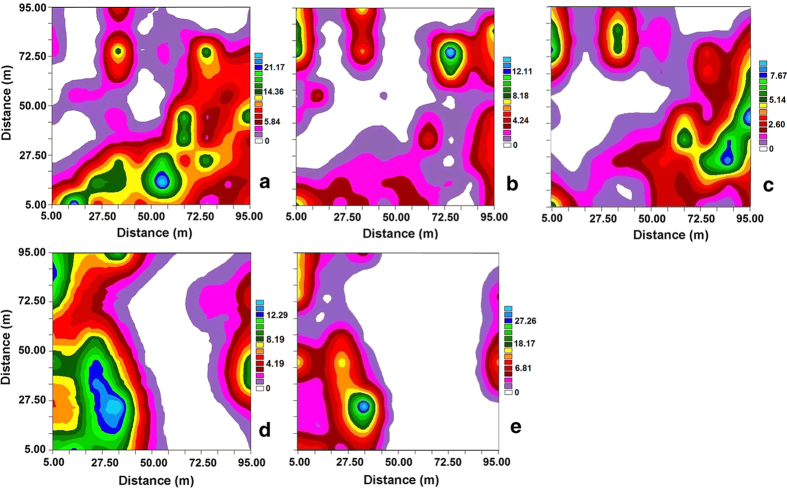
Distribution of 1-year-old seedlings of Chinese yew (*Taxus chinensis*) (**a**) and perching frequency of generalist species (**b**, **c**) vs. specialist species (**d**, **e**) in the patchy habitat of yew ecological garden, Fujian province. (**a**) seedling number of Chinese yew *Taxus chinensis*; (**b**) and (**c**) perching of Red-billed blue magpie *Urocissa erythrorhyncha* in the years of 2011 and 2012 respectively; (**d**) and (**e**) perching of Black Bulbul *Hypsipetes leucocephalus* in the years of 2011 and 2012 respectively. Coloured contours are interpolated from the value of the consponding variable in the centroid of each habitat cell. The color scales are shown.

The seedling census recorded 693 seedlings in the patchy habitat (Fig. 1a), and the seedling distribution pattern was affected by the behaviour of the bird dispersers (Fig. 1b–e).

**Figure 2:**
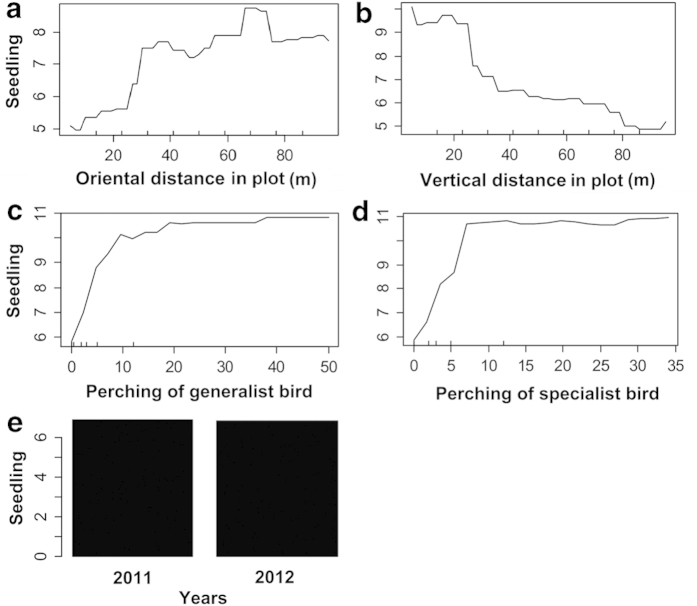
A machine learning algorithm, random forest, for testing the association between the number of seedlings and plot (**a**, **b**), bird perching frequency (**c**, **d**), and years (**e**) in the patchy habitat of yew ecological garden, Fujian province. (**a**) relationship between seedling and oriental distance in plot; (**b**) relationship between seedling and vertical distance in plot; (**c**) relationship between seedling and perching of Red-billed blue magpie *Urocissa erythrorhyncha*; (**d**) relationship between seedling and perching of Black Bulbul *Hypsipetes leucocephalus*; (**e**) relationship between seedling and Sampling years.

After foraging, 512 post-foraging behavioural observations were recorded (284 in 2011 and 228 in 2012) for a forest generalist species (*U. erythrorhyncha*) and 878 post-foraging observations (452 in 2011 and 426 in 2012), for a forest specialist species (*H. leucocephalus*) in the patchy habitat. The generalized linear mixed-effects model indicated that both the generalist and specialist significantly contributed to the number of seedlings (generalized linear mixed-effects model, generalist: *p* = 0.0767; specialist: *p* = 0.0812), whereas the interaction term for the generalist and specialist was not significant (generalized linear mixed-effects model, generalist: specialist, *p* = 0.2255) (Table 3). The random forest results clearly showed a positive association between the birds’ activities and seedling counts; however, the contributions of the specialist and generalist to seedling dispersal did not differ with regard to quality (random forest: 35.72% of seedlings could be explained by five variables) (Fig. 2).

## Discussion

The worldwide concern of habitat loss and fragmentation has led to examination of the quality of seed dispersal service in patchy habitats^2^,^3^. In general, many bird-dispersed species are characterized by a diverse frugivore community in natural habitats^1^. Therefore, attracting similar frugivorous birds to forage and disperse seeds is an important issue for plant persistence in the patchy habitat. In the case of *T. chinensis*, the plant established a seed dispersal system with birds of the Pycnonotidae and Corvidae families (Table 1 and 2), similar to those in other geographical populations in Zhejiang and Anhui^16^,^17^. Stability of disperser species across different regions could facilitate the formation of seed dispersal mutualism in patchy habitats^1^.

High levels of seed removal by frugivorous birds correspond to increased opportunities for plants to colonize new habitat patches, thus enhancing the persistence of plant populations in patch habitats^5^. In the case of *T. chinensis*, frugivorous birds foraged and dispersed more seeds in a patchy habitat (Table 2) than the bird species in Zhejiang and Anhui^16^,^17^, possibly because of aggregation of fruiting trees. This conclusion was also reported by another study of bird-dispersed trees^3^. Moreover, specialist species and generalist species differed in their fruit consumption; more seeds were foraged and dispersed by specialist species than generalist species. Therefore, the contribution of the specialist assemblage to dispersal quantity was higher than that of generalists (Tables 1 and 2), possibly as a result of the frugivorous bird composition and its dietary composition. In our patchy habitat, the frugivore community was dominated by specialist species rather than generalist species (Table 1 and S1), and most of the specialist species were obligate frugivores, whereas some of the generalist species were omnivores^18^. Thus, when the plants are foraged by an assemblage of dominant species or obligate frugivores, this type of disperser assemblage may show better dispersal efficacy with regard to dispersal quantity^4^,^5^.

In general, dispersal quality was affected by the post-behavioural decision of frugivores, which could determine seed deposition patterns and thus affect plant recruitment^13^,^19^,^20^. Often, bird dispersers exhibit complex habitat selection patterns in patchy habitats, affecting their dispersal quality^5^,^11^. In the case of *T. chinensis*, both generalist and specialist species significantly contributed to the number of seedlings and showed similar contributions to seedling recruitment (Table 3; Fig. 2). Therefore, the seed dispersal mutualism of *T. chinensis* in the patchy habitat was dominated by an effective disperser assemblage. This ecological phenomenon occurs not only in patchy habitats but also in other disturbed habitats, such as national parks and botanical gardens, thus indicating increased plant persistence in disturbed habitats^16^,^21^,^22^.

Our results highlight the ability of the *T. chinensis* yew to recruit an effective disperser assemblage in patchy habitats. If both generalist and specialist species could transport the seeds into a suitable site for regeneration, the plants will achieve persistence in disturbed patchy habitats.

## Methods

### Plant species and study site

*T. chinensis* is a dioecious gymnosperm endemic to China that is wind-pollinated and distributed in evergreen broadleaf forests. Female plants bear axillary cones each year that develop into fleshy single-seeded arils in autumn. An average *T. chinensis* tree produces more than 4000 fruits annually^16^,^17^.

The study was conducted in a yew ecological garden (elevation 895–1218 m a.s.l., slope angle 27°), located in the southern experimental area of the Meihua Mountain National Nature Reserve (25° 15′–25° 35′ N; 116° 45′–116° 57′ E) in Fujian province in the southern region of the Wuyi Mountains. This site contains the largest wild *T. chinensis* population in China (approximately 490 adults), including 200 trees that are >500 years old. A specially protected reserve of 15 ha was established by the local government in 2003 to protect this population. The vegetation and the yew population are highly fragmented because of long-term human use. Human-modified patches of bamboo patches and mixed bamboo and broadleaf patches were interlaced with yew patches to form a heterogeneous mosaic (Fig. S1).

### Diversity of forest generalists and specialists

To quantify the diversity of forest generalists and specialists within the *T. chinensis* habitat, we estimated the species richness, diversity, and total abundance of frugivorous birds during the fruiting season using line transects. One transect (50 m wide × 2 km long) was established through the *T. chinensis* population, and two parallel transects of the same dimensions were established 100 m from each side of the first transect. Two investigators walked each transect between 07:00 and 10:00 AM and between 16:00 and 18:00 PM and counted the number of frugivorous birds^23^. Each transect was investigated every three days. In total, the study site was surveyed for 180 hours over 2 years. We retained the highest value of recorded individuals during six censuses for each frugivorous species. Forest generalists and specialists were defined by their habitat adaptation^23^. Then, the Shannon-Wiener index, Pielou species evenness index, and Simpson’s dominance index were used to compare species diversity between generalists and specialists^24^,^25^.

### Quantity of seed dispersal by generalists and specialists

The quantity aspect of dispersal effectiveness (numbers of seeds dispersed) was evaluated by measuring the foraging behaviour of frugivorous birds. Ten mother yew trees were observed during the 2011 and 2012 fruiting seasons (October 20 to December 10). Each tree was observed with binoculars for 8 h starting at sunrise, from a hide located at least 20 m from the tree. The observations ended when no fruit remained on the trees. All observations were made in good weather.

Species, time spent foraging, number of fruits foraged and fruit-handling behaviour were recorded for each bird that visited a mother tree, from the time of its arrival until it left the tree. If a group of conspecific birds visited the tree and the behaviour of all birds could not be observed simultaneously, we focused on the individual that was most visible^26^,^27^. Seed dispersers were defined as bird species that were observed swallowing fruits or carrying fruits away in their beaks^28^. We used *t*-tests to compare the visiting frequency of frugivorous bird species and disperser species between the two years^29^.

### Quality of seed dispersal by generalists and specialists

Previous studies have shown that the seeds of *T. chinensis* are dispersed primarily by Corvidae and Pycnonotidae^16^,^17^,^30^. In our study, we selected two bird species in these two families *H. leucocephalus* vs. *U. erythrorhyncha* with the highest dispersal quantity and different habitat adaptations, and we evaluated the quality aspect of effectiveness by testing the spatial correlation between bird perching frequency and the quantity of 1-year-old seedlings, because seedling numbers and distribution are significantly affected by disperser behaviour in patchy habitats^27^. A strong positive spatial correlation between perching frequency and 1-year-old seedlings indicates that a bird can transport a relatively large number of seeds to seedling habitats, thereby contributing to the quality of dispersal^11^,^27^.

Initially, a plot of 100 m × 100 m was established according to the terrain of study area with focal mature trees as the centre. One hundred 10 × 10-m^2^ habitat cells were used to digitize the plot. One-year-old seedlings (height ≤ 10 cm) were located and recorded in each cell after the fruiting season of 2012. We observed the post-foraging behaviours of the two bird species in 2011 and 2012 respectively, and recorded the habitat cells in which birds perched after they left the study trees. The perching locations of each bird were recorded every 30 s until the bird was lost from sight^27^. Perching locations were used to identify characteristic landmarks near the study trees for later map-based verification of estimates.

The Kolmogorov–Smirnov test was performed using SPSS 19.0 (SPSS, Inc., Chicago, IL) to determine whether data on seedling numbers and perching frequency of birds were normally distributed. The semivariogram function was used to explain the spatial pattern of 1-year-old seedlings and the bird perching pattern. Block kriging was applied to interpolate these variables into a spatial surface map using GS + 3.1^29^. Furthermore, the numbers of seedlings in the habitat cells were explained in a generalized mixed effect model, where the perching frequency of birds (generalist and specialist) were the covariates, and survey year, habitat cells, and their interaction term were the random effects. We used the function glmer in the R Ver. 3.1.2 package “lme4” for the analysis. Because the numbers of seedlings are count data, we selected the Poisson distribution in glmer. Random forest is an ensemble machine-learning method for classification and regression that operates by constructing a multitude of decision trees. It is appropriate for illustrating the nonlinear effect of variables, can handle complex interactions among variables, and is not affected by multicollinearity. Random forest can assess the effects of all explanatory variables simultaneously and automatically ranks the importance of variables in descending order. The partial plot of the explanatory variables provided by R package Random Forest^31^,^32^ can give detailed information regarding the effects of explanatory variables. Therefore, in our study, we used a machine-learning algorithm, random forest, to plot the partial effect of each variable, including the location index representing the spatial locations of the habitat cells.

## Author Contributions

N.L., C.-H.L. and S.-Q.A. designed the study. N.L. S.-B.F. performed data collection. N.L., X.-H. L., S.-B.F. conducted all statistical analyses. All authors contributed to the writing and editing of the manuscript.

## Additional Information

**How to cite this article**: Li, N. *et al*. Differential contribution of frugivorous birds to dispersal patterns of the endangered Chinese yew (Taxus chinensis). *Sci. Rep*. **5**, 10045; doi: 10.1038/srep10045 (2015).

## Supplementary Material

Supplementary figures and tables Supplementary figure1 and table 1.

## Figures and Tables

**Table 1:** Diversity comparison between forest generalists and specialists during the fruiting season of *Taxus chinensis* in the patchy habitat of yew ecological garden, Fujian province.

Measure of diversity	Forest generalists	Forest specialists
Shannon-Wiener diversity	2.65	1.85
Species evenness index	0.79	0.64
Simpson’s dominance index	0.11	0.24

**Table 2:** Frugivorous birds visiting *Taxus chinensis* in the patchy habitat of yew ecological garden, Fujian province.

Bird species	Foraging frequency		Feeding amount	Length of foraging		Habitat adaptive
	2011	2012				
Seed disperser						
* Hypsipetes leucocephalus*	233	253	16.0 ± 14.6	16.2 ± 13.6		S
* Urocissa erythrorhyncha*	22	18	16.3 ± 9.1	18.4 ± 16.4		G
* Copsychus saularis*	7	5	8.0 ± 3.5	11.4 ± 5.5		G
* Dendrocitta formosae*	6	15	19.9 ± 11.5	23.8 ± 11.9		G
* Hemixos castanonotus*	3	22	9.6 ± 9.9	20.6 ± 12.0		S
* Corvus macrorhynchos*	2	X	11.0	20.0		G
* Hypsopetes mcclellandii*;	1	2	12.0 ± 4.0	15.0 ± 5.0		S
* Spizixos semitorques*	1	6	11.3 ± 7.9	27.7 ± 10.1		G
* Garrulax monileger*	1	X	6.0	10.0		S
* Garrulax cineraceus*	1	X	2.0	6.0		S
* Pycnonotus jocosus*	X	19	16.2 ± 14.2	33.3 ± 25.8		G
* Garrulax pectoralis*	X	5	14.4 ± 6.9	22.0 ± 21.4		S
* Harpactes erythrocephalus*	X	1	3.0	5.0		S

Pulp consumer						
* Pericrocotus solaris*	19	6	16.2 ± 14.2	33.0 ± 25.8	S	
* Pericrocotus flammeus*	2	5	14.8 ± 15.6	18.8 ± 10.0	S	
* Alcippe morrisonia*	1	3	22.3 ± 9.3	32.7 ± 23.7	S	
* Yuhina zantholeuca*	X	13	4.1 ± 2.2	25.6 ± 16.9	S	
* Phoenicurus auroreus*	X	4	7.3 ± 2.2	12.0 ± 5.4	G	
* Ficedula albicilla*	X	1	5.0	10.0	S	
* Parus major*	X	5	4.0 ± 1.9	17.4 ± 9.1	G	
* Periparus venustulus*	X	2	7.5 ± 0.5	22.5 ± 2.5	S	
* Sturnus sericeus*	X	1	10.0	25.0	G	
Total	299	368				

Seed dispersers are those birds that swallow entire fruits, defecating or regurgitating the seeds. Pulp consumers are those species that peck the fruit pulp and discard the seed^28^. Unit time is an 8-h period starting at sunrise. Results are presented as the means ± SE. X, Species not recorded. G: generalist, S: specialist.

**Table 3:** A generalized linear mixed-effects model showing the association between seedling numbers and bird activities in 2011 and 2012 in the patchy habitat of yew ecological garden, Fujian province.

Fixed effects				
Factors	Estimate	Std. Error	z value	P-value
Intercept	0.899	0.186	4.835	1.33E-06***
Generalist	0.022	0.013	1.770	0.0767*
Specialist	0.024	0.014	1.744	0.0812*
Generalist: Specialist	−0.002	0.002	−1.212	0.2255
Random effects				
Groups	Variance	Std. Dev.		
Year	2.961e-09	5.442e-05		
Plot	2.468e + 00	1.571e + 00		
Year: Plot	2.769e-08	1.664e-04		

Significance: 0 ‘***’, 0.1 ‘*’
