# Supplementary material for: Differential contribution of frugivorous birds to dispersal patterns of the endangered Chinese yew (Taxus chinensis)
Source: Sci Rep. 2015 May 5;5:10045. doi: 10.1038/srep10045 (PMC4419539; doi:10.1038/srep10045)
Supplement: Supplementary figures and tables — Supplementary figure1 and table 1. [file srep10045-s1.doc]

# Differential contribution of frugivorous birds to dispersal patterns of the endangered Chinese yew (*Taxus chinensis*)

Ning Li^1,2^, Shu-bo Fang^2,3^, Xin-hai Li^4^, Shu-qing An^2^ & Chang-hu Lu^1^,*

**Figure S1 Patch composition in the yew ecological garden, Fujian Province, southeast China**

**
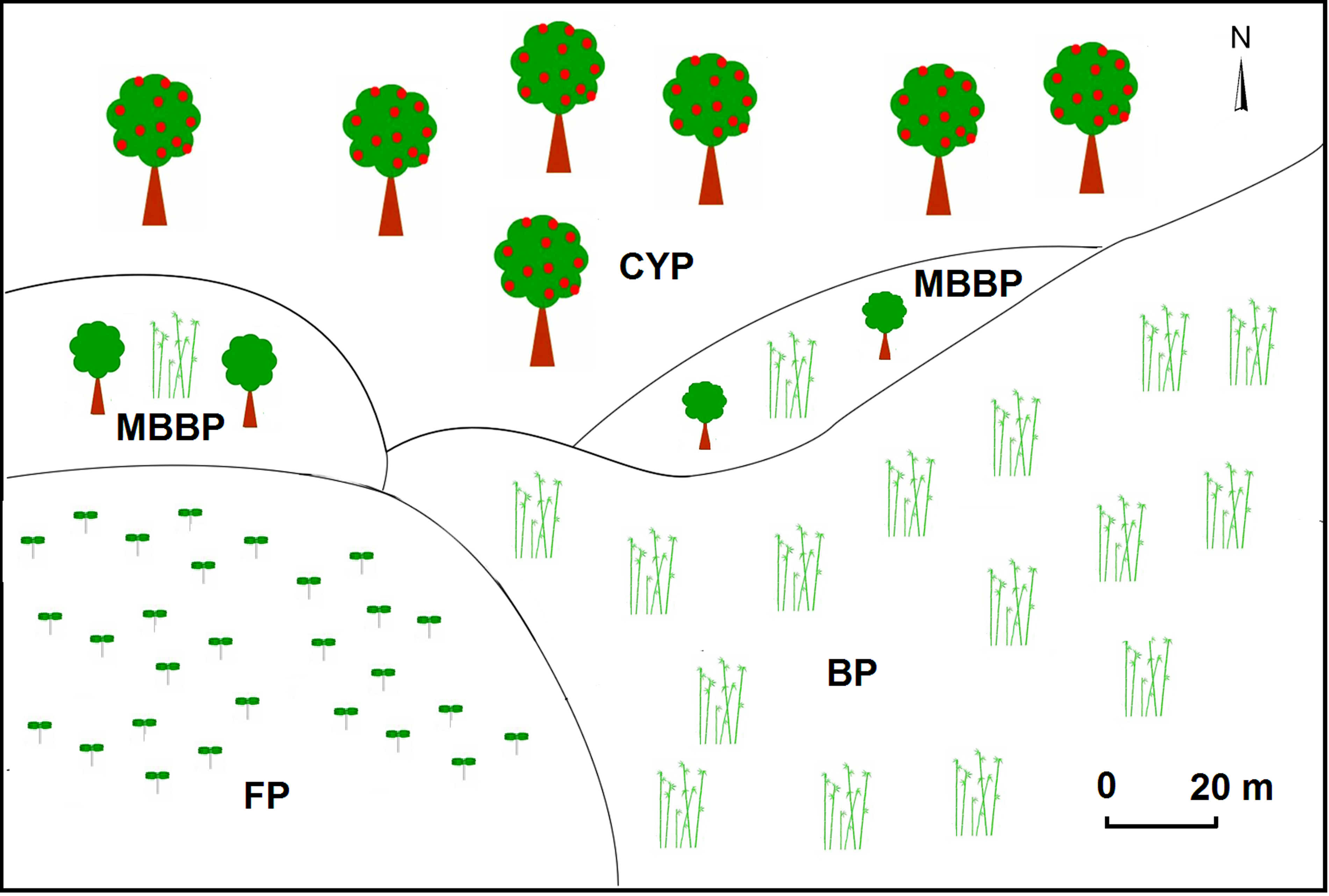
**

Patch type: BP, Bamboo patch; CYP, Chinese yew patch; FP, Farmland patch; MBBP, Mixed bamboo and broadleaf patch

**Table S1** Forest generalist and specialist observed in the neighborhood of ten study trees in the fruiting season of 2011 and 2012

| Order | Family | Species | Individuals |
| --- | --- | --- | --- |
| Forest generalist | | | |
| Galiformes | Phasianidae | *Bambusicola thoracica* | 3 |
|  |  | *Lophura nythemera* | 7 |
| Cuculiformes | Centropdidae | *Centropus bengalensis* | 1 |
|  | Cuculidae | *Polophilus sinensis* | 1 |
| Galbulidae | Picidae | *Picus canus* | 2 |
| Passeriformes | Nectariniidae | *Aethopyga christinae* | 5 |
|  | Motacillidae | *Anthus hodgsoni* | 4 |
|  |  | *Anthus sylvanus* | 4 |
|  | Turdidae | *Copsychus saularis* | 5 |
|  |  | *Enicurus leschenaulti* | 1 |
|  |  | *Myophonus caeruleus* | 2 |
|  |  | *Phoenicurus auroreus* | 30 |
|  | Corvidae | *Corvus macrorhynchos* | 1 |
|  |  | *Dendrocitta formosae* | 15 |
|  |  | *Urocissa erythrorhyncha* | 76 |
|  | Emberizidae | *Emberiza cioides* | 3 |
|  |  | *Emberiza spodocephala* | 8 |
|  | Timaliidae | *Leiothrix lutea* | 25 |
|  | Ploceidea | *Lonchura punctulata* | 5 |
|  |  | *Lonchura striata* | 40 |
|  | Paridae | *Parus major* | 12 |
|  | Passeridae | *Passer montanus* | 20 |
|  | Pycnonotidae | *Pycnonotus aurigaster* | 2 |
|  |  | *Pycnonotus jocosus* | 12 |
|  |  | *Spizixos semitorques* | 12 |
|  | Muscicapidae | *Tarsiger cyanurus* | 4 |
|  |  | *Rhyacornis fuliginosus* | 2 |
|  | Sturnidae | *Sturnus sericeus* | 2 |
| Forest specialist | | | |
| Cuculiformes | Trogonidae | *Harpactes erythrocephalus* | 6 |
| Galbulidae | Capitonidae | *Megalaima virens* | 1 |
| Passeriformes | Passeriformes | *Chloropsis hardwickii* | 1 |
|  | Timaliidae | *Garrulax cineraceus* | 5 |
|  |  | *Garrulax pectoralis* | 30 |
|  | Corvidae | *Garrulus glandarius* | 2 |
|  | Pycnonotidae | *Hemixos castanonotus* | 15 |
|  |  | *Hypsipetes leucocephalus* | 100 |
|  |  | *Hypsipetes mcclellandii* | 5 |
|  | Paridae | *Parus spilonotus* | 4 |
|  |  | *Periparus venustulus* | 1 |
|  | Campephagidae | *Pericrocotus flammeus* | 10 |
|  |  | *Pericrocotus solaris* | 100 |
|  | Sylviidae | *Yuhina zantholeuca* | 5 |
|  | Muscicapidae | *Alcippe morrisonia* | 10 |
|  |  | *Ficedula parva* | 1 |
|  |  | *Garrulax monileger* | 3 |
|  |  | *Zoothera dauma* | 1 |
